# Glucocorticoid receptors in the locus coeruleus mediate sleep disorders caused by repeated corticosterone treatment

**DOI:** 10.1038/srep09442

**Published:** 2015-03-24

**Authors:** Zi-Jun Wang, Xue-Qiong Zhang, Xiang-Yu Cui, Su-Ying Cui, Bin Yu, Zhao-Fu Sheng, Sheng-Jie Li, Qing Cao, Yuan-Li Huang, Ya-Ping Xu, Yong-He Zhang

**Affiliations:** 1 Department of Pharmacology, Peking University, School of Basic Medical Science, Beijing 100191, China

## Abstract

Stress induced constant increase of cortisol level may lead to sleep disorder, but the mechanism remains unclear. Here we described a novel model to investigate stress mimicked sleep disorders induced by repetitive administration of corticosterone (CORT). After 7 days treatment of CORT, rats showed significant sleep disturbance, meanwhile, the glucocorticoid receptor (GR) level was notably lowered in locus coeruleus (LC). We further discovered the activation of noradrenergic neuron in LC, the suppression of GABAergic neuron in ventrolateral preoptic area (VLPO), the remarkable elevation of norepinephrine in LC, VLPO and hypothalamus, as well as increase of tyrosine hydroxylase in LC and decrease of glutamic acid decarboxylase in VLPO after CORT treatment. Microinjection of GR antagonist RU486 into LC reversed the CORT-induced sleep changes. These results suggest that GR in LC may play a key role in stress-related sleep disorders and support the hypothesis that repeated CORT treatment may decrease GR levels and induce the activation of noradrenergic neurons in LC, consequently inhibit GABAergic neurons in VLPO and result in sleep disorders. Our findings provide novel insights into the effect of stress-inducing agent CORT on sleep and GRs' role in sleep regulation.

Stress triggers important neuroendocrine responses that enable the organism to survive and restore homeostasis. The primary responses include the rapid activation of hypothalamic–pituitary–adrenal (HPA) axis and sympathoadrenal system, leading to the release of adrenocorticotropic hormone, glucocorticoids, and catecholamines^1^. Sleep is an important component of human homeostasis. Sleep disorders are closely associated with significant medical, psychological, and social disturbances, such as depression^2^. Activation of the HPA axis or sympathetic nervous system results in wakefulness, and these hormones, including corticotropin-releasing factor, adrenocorticotropic hormone, cortisol (or corticosterone; CORT), norepinephrine, and epinephrine, are associated with attention and arousal^3^.

Glucocorticoids are the final mediators in HPA axis cascade and critical for the pathogenesis of sustained stress-related sleep disorders. Many researchers have reported that sustained stress^4^,^5^ increases cortisol levels and can induce sleep disorders, including poor sleep quality and shorter sleep duration. Corticosteroid receptors, glucocorticoid receptors (GRs) and mineralocorticoid receptors (MRs), are highly expressed in the brain^6^. Researchers have demonstrated that glucocorticoids can regulate sleep directly via MRs and GRs^7^,^8^. Because of the different affinities of these two receptors, GRs play an important role when corticosteroids reach stressful levels^1^. Additionally, GRs are abundantly expressed in sleep-wake-related brain stem nuclei^9^,^10^.

The sleep-wake-related brain nuclei contain sleep-promoting γ-aminobutyric acid (GABA) neurons in ventrolateral preoptic nucleus (VLPO), and wake/active neurons including histaminergic tuberomammillary nucleus (TMN), orexinergic perifornical area (Pef), serotonergic dorsal raphe nuclei (DRN), noradrenergic locus coeruleus (LC), and cholinergic neurons in the pontine laterodorsal and pedunculopontine tegmental (LDT/PPT) nuclei^11^,^12^. Retrograde and anterograde tract-tracing studies indicated that VLPO neurons are reciprocally connected with TMN, Pef, DRN, LC and LDT/PPT^13^,^14^. Cano *et al*.^15^ reported that acute stress-induced insomnia was related to altered activity in sleep-wake regulating nuclei such as VLPO, LC. In addition, both acute stress (such as footshock or immobilization^16^,^17^) and acute hydrocortisone administration^18^ have been shown to suppress sleep in animal models. In the pilot studies, we demonstrated that repeated administration of glucocorticoids induced sleep changes were different. Thus, the unknown mechanism of repeated stress-induced sleep disorders needs further investigation.

To better understand the underlying mechanism of long-term treatment of glucocoritcoids induced sleep disorders, we repeatedly treated rats with CORT for 7 days to mimic stress, and these animals were then evaluated with regard to sleep parameters. To correlate such behavior with possible underlying mechanisms, we measured the activity of sleep-wake-regulating nuclei, major monoamine neurotransmitters in related brain areas, GR and MR expression in LC, glutamic acid decarboxylase (GAD) in VLPO, and tyrosine hydroxylase (TH) in LC. The levels of GRs decreased in LC after 7-day CORT treatment, prompting us to test whether administration of the GR antagonist RU486 can reverse stress-related sleep disturbances.

## Results

### Repeated administration of CORT induced sleep disorders in rats

Table 1 showed that there were significant reduction of total sleep time (TST, *F*_1,16_ = 36.987, *p* < 0.001), non-rapid eye movement sleep time (NREM, *F*_1,16_ = 55.206, *p* < 0.001) and light sleep time (LST, *F*_1,16_ = 36.624, *p* < 0.001) after CORT treatment; While rapid eye movement sleep time (REM) was obviously increased compared with vehicle (*F*_1,16_ = 6.086, *p* = 0.026), and slow wave sleep time (SWS, *F*_1,16_ = 0.950, *p* = 0.345) was unaffected. Meanwhile, the administration of CORT significantly prolonged the sleep latency (SL, *F*_1,16_ = 16.672, *p* = 0.001) and shortened the REM sleep latency (REM SL, *F*_1,16_ = 23.114, *p* < 0.001). The REM sleep time ratio (REM%, *F*_1,16_ = 13.980, *p* = 0.002) was significantly raised up as well without changing of light sleep time ratio (LST%, *F*_1,16_ = 1.613, *p* = 0.223) and SWS time ratio (SWS%, *F*_1,16_ = 0.436, *p* = 0.519).

### Repeated administration of CORT augmented neuronal activity in LC and suppressed neuronal activity in VLPO

**Figure 1:**
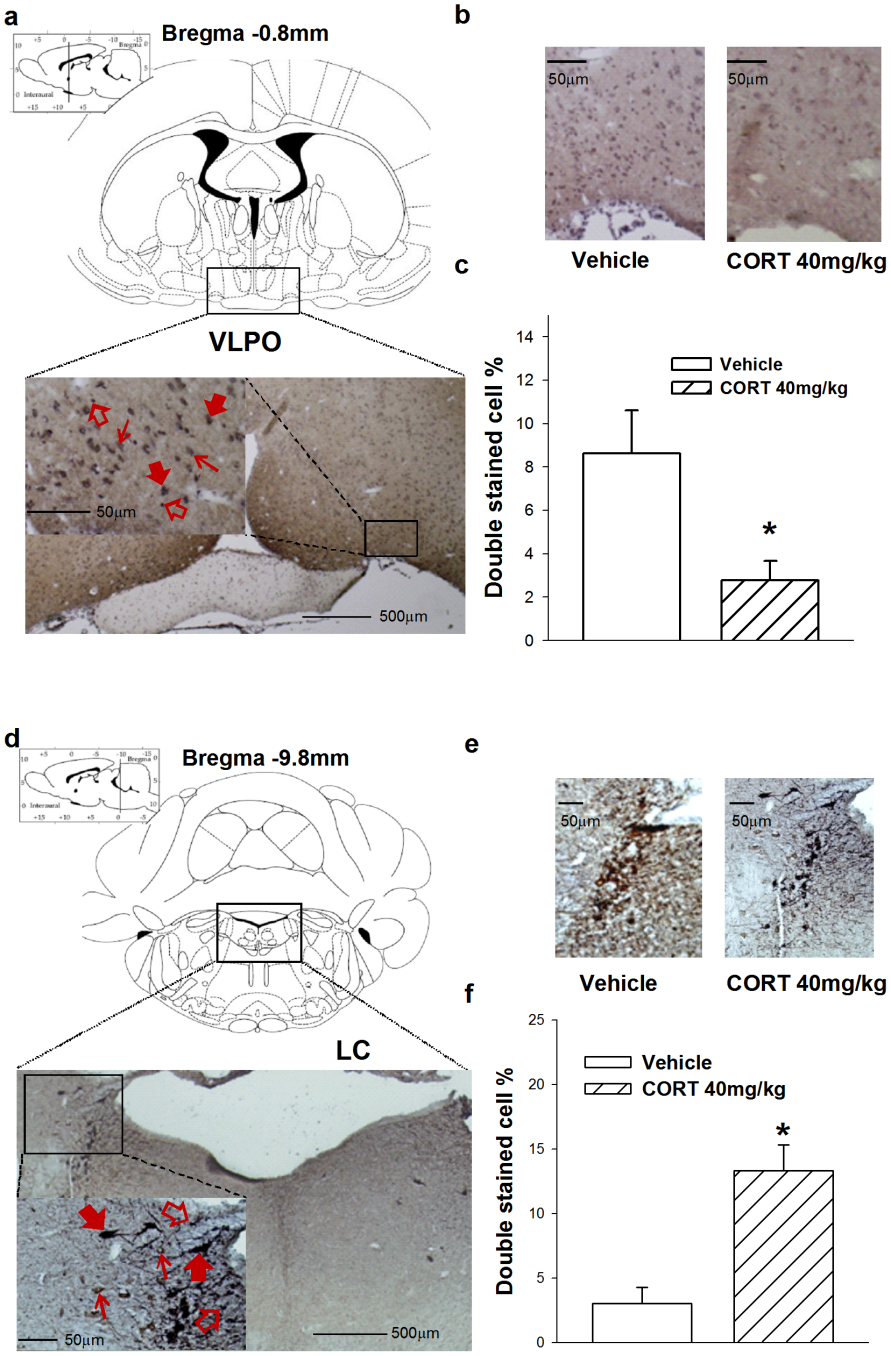
Treatment of CORT (40 mg/kg, s.c.) for 7 days activated noradrenergic neuron in the LC and suppressed GABAergic neuron activity in the VLPO. Representative and quantification of GAD+ and Fos+ double stained cell ratio in VLPO (b, c) as well as TH+ and Fos+ double stained cell ratio in LC (e, f) was calculated. Data are represented as mean ± S.E.M. (*n* = 5 ~ 6/group, *p < 0.05 and **p < 0.01 *vs* vehicle). Representative photographs of immunostained sections showing the cytoarchitectonic characteristics observed in the VLPO (a) and LC (d). Solid red arrows indicate double-labeled neurons; thin arrows indicate a neuron-immunopositive cell; hollow arrows indicate a Fos immunopositive cell. (CORT: Corticosterone; VLPO: Ventrolateral preoptic area; GAD: Glutamic acid decarboxylase; TH: Tyrosine hydroxylase).

**Figure 2:**
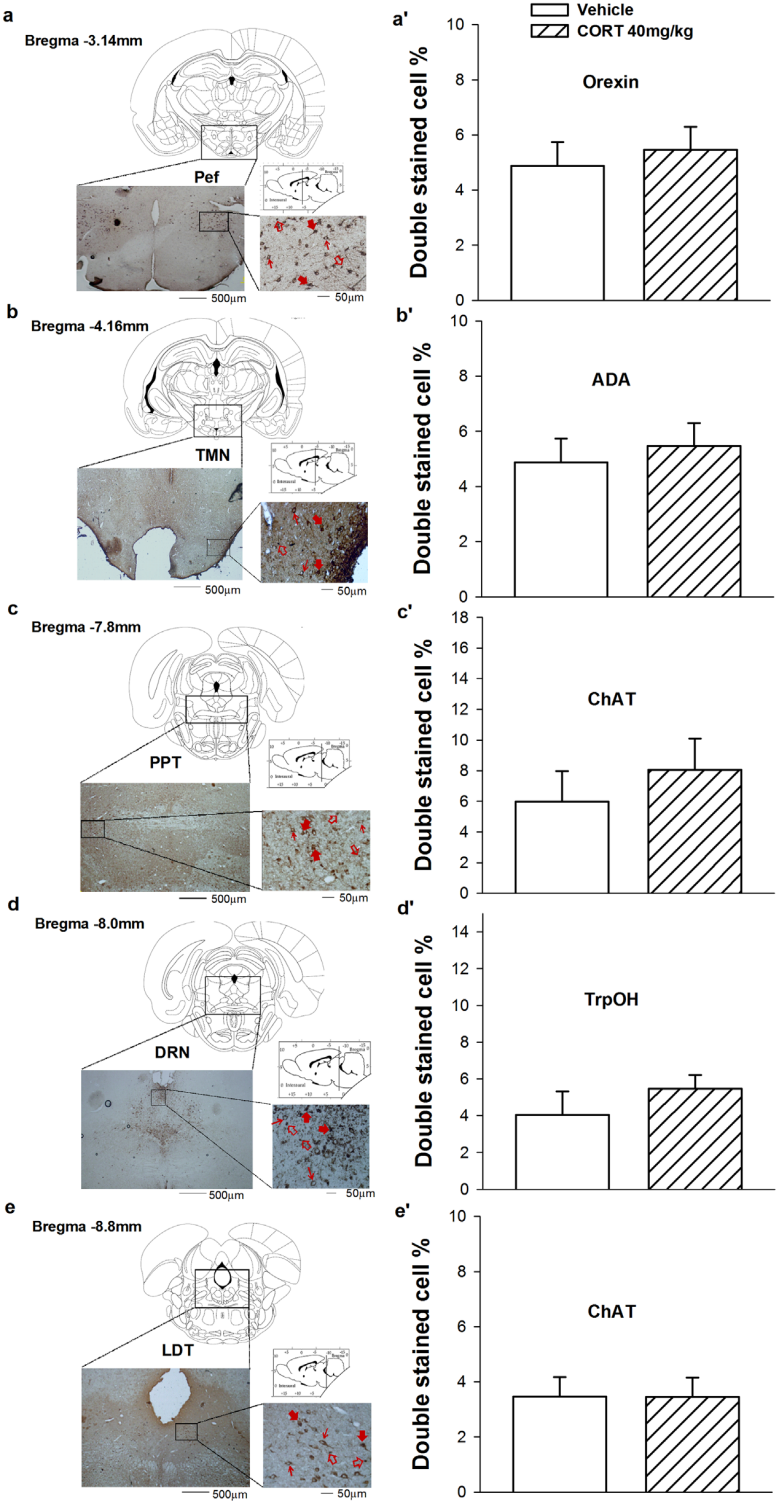
Treatment of CORT (40 mg/kg, s.c.) for 7 days didn't affect the neuronal activity in the Pef, TMN, PPT, DRN and LDT. Quantification of Orexin+ and Fos+ double stained cell ratio in Pef (a′), ADA+ and Fos+ double stained cell ratio in TMN (b′), ChAT+ and Fos+ double stained cell ratio in PPT (c′) and LDT (e′), as well as TrpOH+ and Fos+ double stained cell ratio in DRN (d′) were calculated. Data are represented as mean ± S.E.M. (*n* = 5 ~ 6/group, *p < 0.05 and **p < 0.01 *vs* vehicle). Representative photographs of immunostained sections showing the cytoarchitectonic characteristics observed in the Pef (a), TMN (b), PPT (c), DRN (d) and LDT (e). Solid red arrows indicate double-labeled neurons; thin arrows indicate a neuron-immunopositive cell; hollow arrows indicate a Fos immunopositive cell. (ADA: Adenosine deaminase; ChAT: Choline acetylase; TrpOH: Tryptophan hydroxylase; Pef: perifronical area; TMN: Tuberomammillary nucleus; PPT: Pedunculopontine tegmental nucleus; DRN: Dorsal raphe nucleus; LDT: Laterodorsal tegmental nucleus).

To identify alterations in sleep-wake-related nuclei, we performed double-staining immunohistochemistry in the VLPO, Pef, TMN, PPT, DRN, LDT, and LC. After 7 days CORT treatment (40 mg/kg, s.c.), the ratio of Fos+ and GAD+ neurons in the VLPO (Fig. 1c) was significantly decreased (*t*_10_ = 2.694, *p* = 0.023), whereas the ratio of Fos+ and TH+ neurons in the LC (Fig. 1f) was significantly increased (*t*_9_ = −2.803, *p* = 0.021). We did not detect noticeable differences (Fig. 2) in the Pef (*t*_10_ = −1.369, *p* = 0.201), TMN (*t*_11_ = 0.151, *p* = 0.882), PPT (*t*_10_ = −0.719, *p* = 0.489), DRN (*t*_8_ = −0.974, *p* = 0.359), or LDT (*t*_9_ = 0.019, *p* = 0.985). These results indicate that changes in sleep parameters caused by sustained elevations of CORT involved alterations in neuron activity in the LC and VLPO.

### GR protein level in LC was lowered after repeated administration of CORT

To determine whether the increase in noradrenergic neuron activity in the LC was directly attributable to CORT administration, we analyzed total GR protein levels and GR distribution in the cytoplasm/nucleus and MR protein levels in the LC. Acute CORT administration (1-day CORT administration [CORT 1D group]; i.e., acute CORT administration on day 7 proceeded by 6 days of vehicle administration) was used as another control.

**Figure 3:**
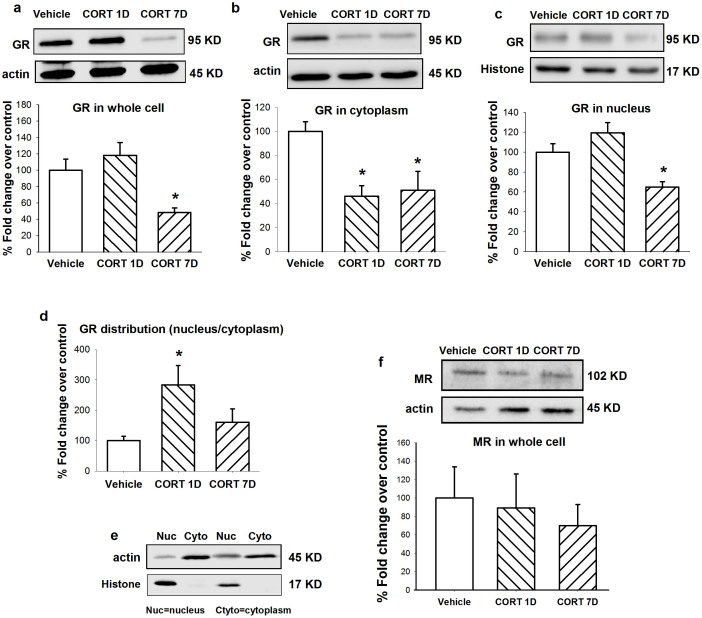
Treatment of 7 days CORT (40 mg/kg, s.c.) decreased GR, but not MR protein levels in the LC. Representative of western blots and quantification of total GR (a), cytoplasm GR (b), nucleus GR (c) and total MR (f) in the LC. (d) The distribution of GR in nucleus/cytoplasm after last injection of CORT. CORT 1D: CORT 40 mg/kg s.c. at day 7 proceeded by 6-day vehicle. (e) β-actin (actin) and histone-3 (histone) are shown as quantitative loading control for cytoplasm and nucleus respectively. Data are represented as mean ± S.E.M. (*n* = 6 pooled samples and repeated for 3 ~ 4 times, *p < 0.05 and **p < 0.01 *vs* vehicle).

After 7 days of CORT treatment (40 mg/kg, s.c.), total GR protein levels in the LC (Fig. 3a) significantly decreased (*F*_2,11_ = 8.489, *p* = 0.019). Specifically, GR protein levels in the cytoplasm (Fig. 3b; *F*_2,11_ = 6.894, *p* = 0.015) and nucleus (Fig. 3c; *F*_2,11_ = 10.760, *p* = 0.004) significantly decreased. The normal distribution of GRs after acute exposure to CORT transferred from the cytoplasm to the nucleus, similar to the CORT 1D group (Fig. 3d; *F*_2,11_ = 4.180, *p* = 0.052). However, no difference was found between the chronic CORT-treated group (CORT 7D) and vehicle group (Fig. 3d). No significant changes in MRs (*F*_2,8_ = 0.226, *p* = 0.804) were observed in the LC (Fig. 3f). These results suggest that GRs in the LC might be mainly involved in mediating the sleep disturbances caused by CORT. The method of separately extracting protein from the cytoplasm and nucleus was verified in Fig. 3e.

Because the highest density of GR expression was found in the LC and not in the VLPO^9^, we predicted that changes in GABAergic neurons in the VLPO may be attributable to the noradrenergic projection from the LC, which inhibited GABAergic neuron activity (see the HPLC results and discussion below).

### Microinjection of RU486 into LC reversed the sleep disruptions induced by repeated administration of CORT

**Figure 4:**
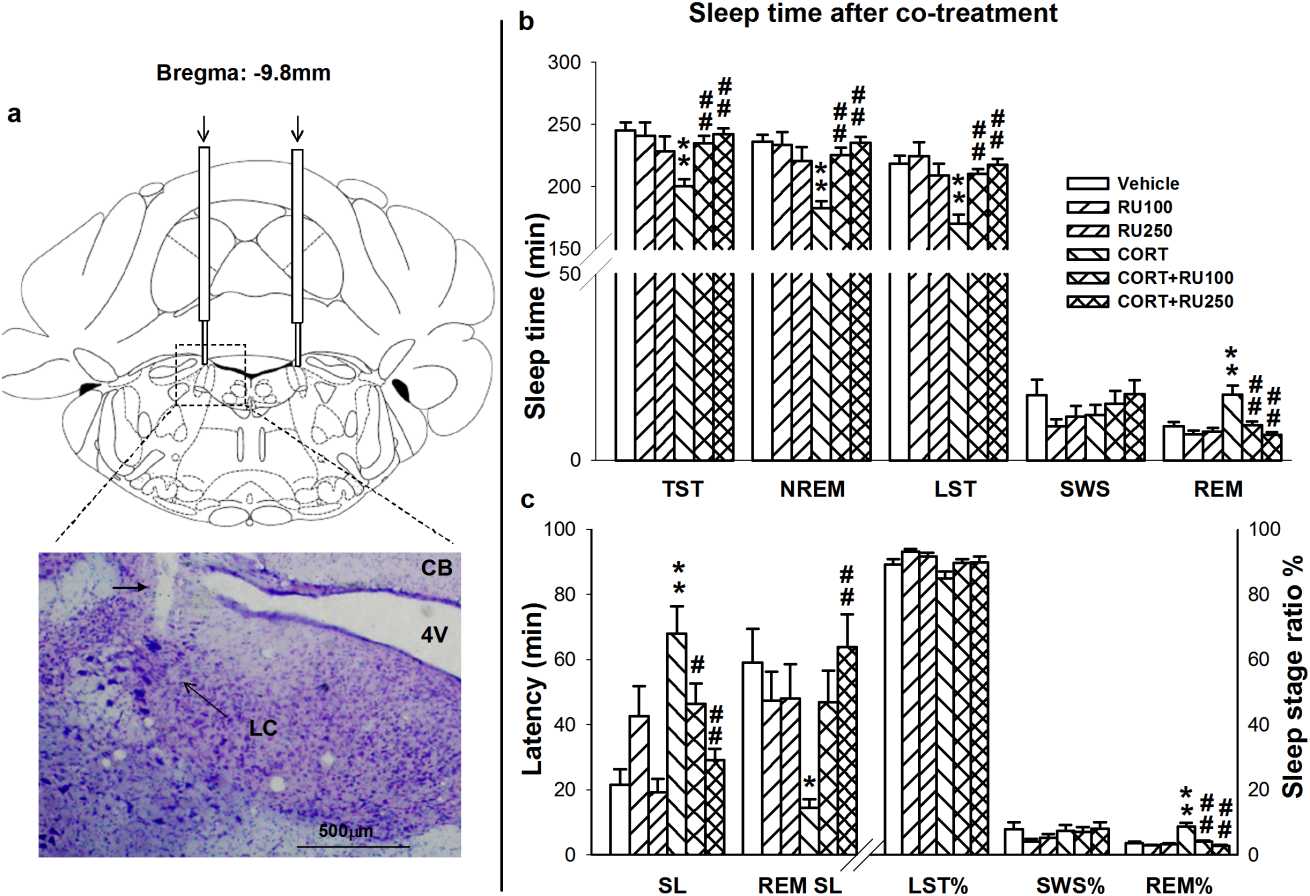
Microinjection of RU486 into the LC reversed CORT induced sleep disorders. (a) The schematic representation of the implantation sites for location of guide cannula 1 mm above the LC and bright-field photomicrograph of a coronal brain section showing the injector tip (black arrow head) for microinjection (4V: fourth ventricle; CB: cerebellum). (b) Sleep parameters including total sleep time (TST), NREM sleep, light sleep time (LST), SWS, REM sleep, and (c) sleep latency (SL), REM SL, light sleep ratio (LST%), SWS%, REM% in total sleep were assessed. RU100 and RU250: RU486 100 ng/side and 250 ng/side (everyday 30 min prior to CORT treatment for 7 days); CORT: 7 days treatment of CORT (40 mg/kg, s.c.). Data are expressed as mean ± S.E.M (*n* = 7 ~ 8/group, *p < 0.05 and **p < 0.01 *vs* Vehicle; # *p* < 0.05 and ##*p* < 0.01 *vs* CORT).

To demonstrate that GR function in the LC is directly related to sleep, we microinjected the GR antagonist RU486 into the LC every day, 30 min prior to CORT administration, to test whether it can antagonize the effect of CORT on sleep. As shown in Fig. 4b, significant decreases in TST (*F*_5,44_ = 4.778, *p* = 0.002), NREM sleep time (*F*_5,44_ = 7.892, *p* < 0.001) and LST (*F*_5,44_ = 7.246, *p* < 0.001) were observed after CORT administration (40 mg/kg, s.c.), which could be dose-dependently reversed by RU486 (100 and 250 ng). Slow wave sleep time was unaffected (*F*_5,44_ = 1.053, *p* = 0.401). Rapid-eye-movement sleep time was increased by CORT treatment and dose-dependently antagonized by microinjection of RU486 (100 and 250 ng) into the LC (*F*_5,44_ = 8.977, *p* < 0.001). As shown in Fig. 4c, RU486 significantly inhibited the CORT-induced prolongation of SL (*F*_5,44_ = 8.544, *p* < 0.001). The CORT-induced decrease in REM SL returned to normal after pretreatment with RU486 (*F*_5,44_ = 3.892, *p* = 0.006). The REM sleep time ratio (REM%) was increased by CORT treatment and significantly decreased by RU486 (*F*_5,44_ = 14.590, *p* < 0.001). No significant difference was found in the LST ratio (LST%; *F*_5,44_ = 3.059, *p* = 0.020) or SWS time ratio (SWS%; *F*_5,44_ = 0.909, *p* = 0.485). These results indicate that pretreatment with RU486 in the LC ameliorated the sleep disturbances caused by repeated CORT administration.

### Repeated administration of CORT increased TH protein in LC and decreased GAD protein in VLPO

**Figure 5:**
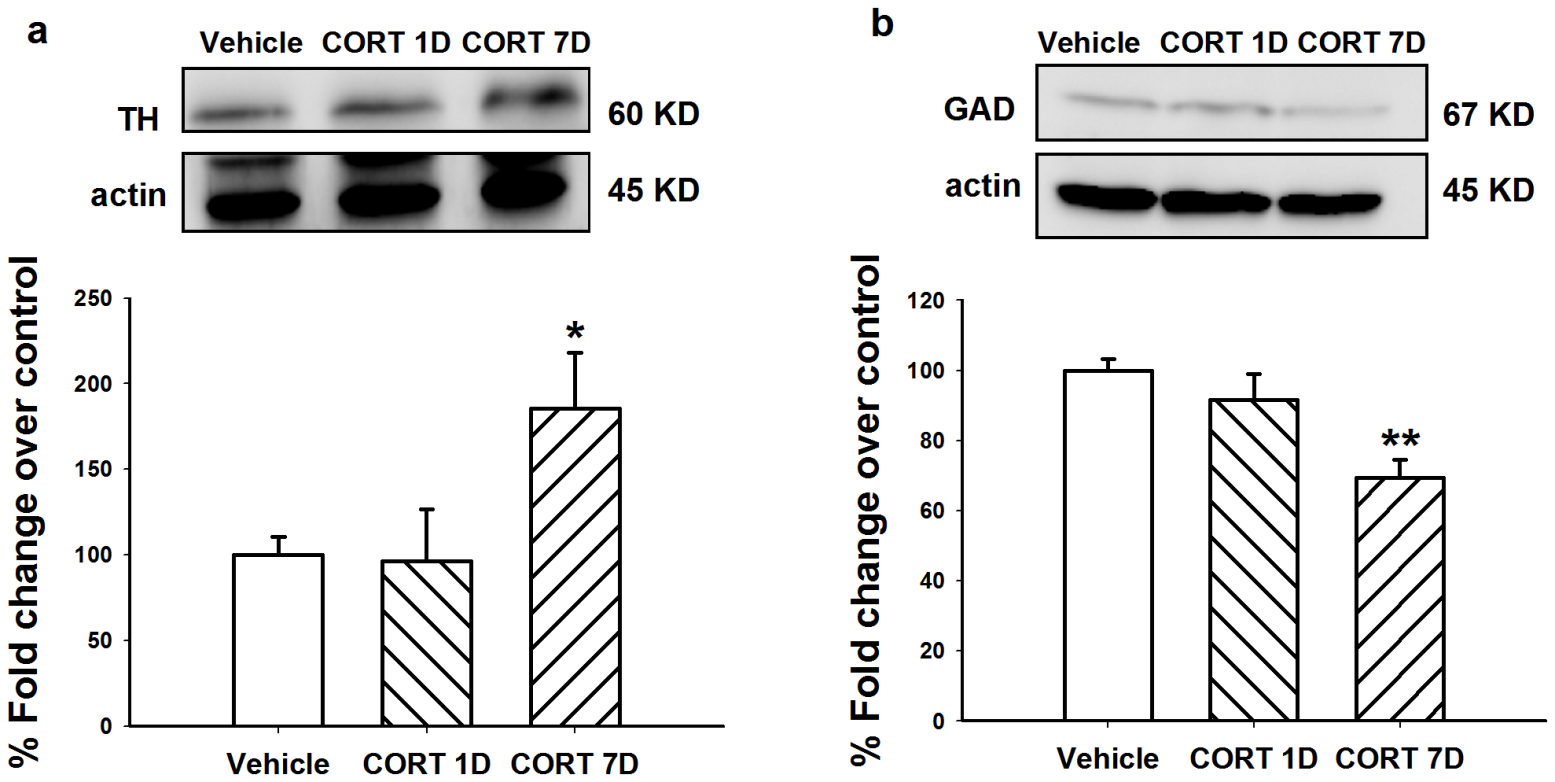
Treatment of CORT (40 mg/kg, s.c.) for 7 days increased TH protein levels in the LC and decreased GAD protein levels in the VLPO. Representative of western blots and quantification of TH (a) in the LC and GAD (b) in the VLPO were shown. CORT 1D: CORT 40 mg/kg s.c. at day 7 proceeded by 6-day vehicle. β-actin (actin) is shown as a quantitative loading control. Data are represented as mean ± S.E.M. (*n* = 6 pooled samples and repeated for 3 ~ 4 times, *p < 0.05 *vs* vehicle).

To validate that repeated GR activation activates TH, Western blot was performed to further verify the changes in total TH and GAD protein levels in the LC and VLPO, respectively. A control group (CORT 1D) was included that received acute CORT administration on day 7 proceeded by 6 days of vehicle administration. After 7 days CORT treatment (40 mg/kg, s.c.), TH protein levels in the LC (Fig. 5a) significantly increased (*F*_2,11_ = 5.771, *p* = 0.024). The protein levels of GAD (Fig. 5b) were significantly decreased in the VLPO (*F*_2,8_ = 8.077, *p* = 0.020).

### Levels of NE and DA in VLPO, LC, hypothalamus and prefrontal cortex after repeated administration of CORT

**Figure 6:**
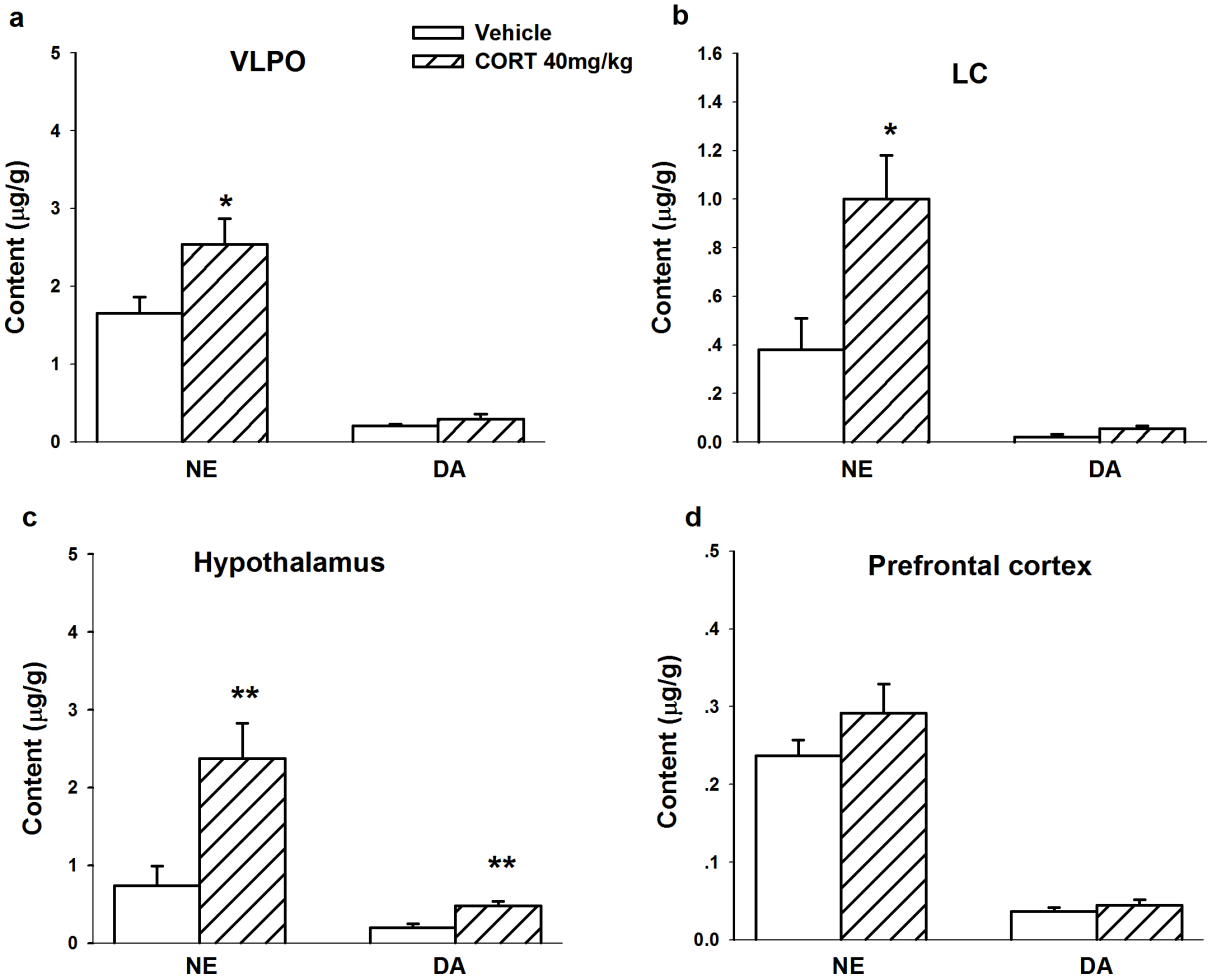
Elevated NE and DA levels in the LC, VLPO and hypothalamus after 7 days treatment of CORT (40 mg/kg, s.c.). NE and DA levels in the VLPO (a), LC (b), hypothalamus (c) and prefrontal cortex (d) were analyzed. Data are calculated as μg/g protein and expressed as mean ± S.E.M. (*n* = 5 ~ 6/group, *p < 0.05 and **p < 0.01 *vs* vehicle).

The results reported above indicate that the noradrenergic system was activated, and we further detected norepinephrine and dopamine levels in related brain areas. The results showed that the levels of norepinephrine in the VLPO (Fig. 6a) were significantly elevated in the CORT-treated group compared with the vehicle group (*F*_1,11_ = 5.046, *p* = 0.048), confirming our hypothesis that increases in norepinephrine in the VLPO inhibit GABAergic neurons. Corticosterone treatment did not alter dopamine levels in the VLPO (*F*_1,11_ = 2.837, *p* = 0.123). In the LC (Fig. 6b), norepinephrine levels were elevated in the CORT-treated group (*F*_1,10_ = 8.175, *p* = 0.019), whereas dopamine levels were unaltered (*F*_1,10_ = 3.726, *p* = 0.086).

The LC is the major source of noradrenergic neurons that sustain a waking state. The noradrenergic projection that passes through the hypothalamus to the cortex is important in the sleep-wake cycle. To verify that noradrenergic neurons in the LC were activated, we detected norepinephrine and dopamine levels in the hypothalamus and prefrontal cortex. The results indicated that the levels of norepinephrine (*F*_1,11_ = 10.004, *p* = 0.010) and dopamine (*F*_1,11_ = 13.597, *p* = 0.004) in the hypothalamus were significantly increased (Fig. 6c) after CORT treatment (40 mg/kg, s.c.). The increases in norepinephrine (*F*_1,9_ = 1.569, *p* = 0.246) and dopamine (*F*_1,9_ = 0.815, *p* = 0.393) in the PFC did not reach statistical significance (Fig. 6d).

## Discussion

In the present study, we used repeated CORT treatment to induce relatively stable and high CORT levels (data not shown) to mimic stress-induced sleep disorders. Under this dosing regimen, we found that rats exhibited significant altered sleep parameters. Immunohistochemistry indicated that repeated CORT administration activated noradrenergic neurons in LC and suppressed GABAergic neurons in VLPO. The activity of other sleep-wake-regulating nuclei, such as the Pef, TMN, PPT, DRN and LDT, were unaffected. The sleep-wake-regulating pathway in the brain^12^ is based on the hypothesis that VLPO is the primary sleep-promoting nucleus that inhibits the TMN, DRN and LC. Insomnia that results from acute stress is related to altered activity in VLPO, TMN and LC^15^. Consistent with this hypothesis, our results suggest that repeated CORT treatment induces insomnia by activating LC and suppressing VLPO.

Lussier *et al*^19^ reported that 14 and 21 days injection of CORT (40 mg/kg) produced clear behavioral changes in the forced swimming test, but 7 days injection of CORT (40 mg/kg) did not show significant immobility compared to vehicle rats. They also proposed that treatment with CORT increases depression-like behavior gradually over a 21-day period and the development of depression-like behavior in CORT treated rats occurs in a linear manner. Our previous report also indicated that after 7 days treatment of CORT (40 mg/kg) rats showed higher level of immobility and lower levels of sucrose preference than did the vehicle rats, but the changes did not reach statistical significance^20^. Based on these reports, it could be presumed that administration of CORT (40 mg/kg) for 7 days may cause the stress-induced abnormal sleep pattern.

Repeated exposure to stress is usually regarded as the main cause of sleep disorders, such as insomnia^21^. Many human studies have linked prolonged stress to self-reported impairments in sleep. For example, sustained stress caused by academic demands^4^ or work shifts^5^ increases corticoid levels and induces sleep disorders, including poor sleep quality, prolonged sleep latency, a shorter sleep duration and disinhibition of REM sleep. In other situations, e.g., depression^22^, mania^23^, schizophrenia^24^, bipolar disorder^25^ or who receive high-dose glucocorticoid treatment for multiple sclerosis^26^,^27^, the sleep disruptions are also observed. Notably, stress-induced sleep disorders are always associated with an increased risk for other psychiatric problems, such as depression^20^,^28^ or panic disorder^29^. The aforementioned evidence indicates that a prolonged increase in corticoid levels caused by stress, disease, or high-dose corticoid treatment can result in sleep disorders. The sleep disorder characteristics of our model in the present study are consistent with previous reports that used other stress-inducing methods^30^,^31^. Thus, the present model may be used to study the underlying mechanism of sleep disorders caused by sustained elevations of corticoid levels.

Corticosteroids are important mediators of homeostasis and stress and exert their complex effects via two receptors, MRs and GRs. Both receptors are expressed in neurons and glia cells in the mammalian brain and contribute to the regulation of brain-cell properties in a slow and persistent manner by modifying the transcription of response genes and *de novo* protein synthesis^1^. The noradrenergic LC abundantly expresses GRs and MRs^9^,^10^. The LC has been found to be a highly stress-reactive nucleus that is capable of prolonged activation and modulating the central stress response^32^,^33^. To confirm whether GRs or MRs in LC participate in sleep disorders induced by repeated CORT administration, we performed two additional experiments. The levels of GRs but not MRs were decreased in LC after 7-day CORT treatment. These results are consistent with the study by Makino *et al*.^34^, which showed that systemic CORT treatment for 1 or 2 weeks decreased GR mRNA in LC. We also directly tested the hypothesis that repeated CORT administration induces sleep disorders by activating GRs in LC. We injected the GR antagonist RU486 directly into LC and as predicted, this treatment resulted in the restoration of normal sleep pattern in CORT-treated rats. Our results are consistent with other studies reported that GR antagonist exerted a sleep-restoring effect in chronic insomnia patients^35^ but not in healthy volunteers^36^,^37^.

Glucocorticoid receptors, after binding glucocorticoids in the cytoplasm, are translocated to the nucleus and bind glucocorticoid response elements (GREs) to activate or repress gene transcription^38^,^39^. Markey *et al*.^40^ indicated that noradrenergic neurons in LC might be target cells for glucocorticoids, meaning that the effect of glucocorticoids on TH might be receptor-mediated. Indeed, excessive GR activation by repeated stress increases TH mRNA^34^,^41^ and TH immunoreactivity^40^,^42^ in LC. Additionally, other studies found a decrease in GAD protein levels after repeated CORT administration in the hippocampus and amygdala^43^. In the present study, we found that repeated CORT administration increased TH levels and TH immunoreactivity in LC as well as decreased GAD levels and GAD immunoreactivity in VLPO.

Stress-induced increases in arousal are related to the noradrenergic system in the brain^44^. The noradrenergic projection from LC is known to promote the waking state in the hypothalamus^45^. Reinforced noradrenergic activation via α1-adrenoceptors in the prefrontal cortex in rats has been found to enhance cognitive performance, and this was interpreted as resulting from an increase in arousal^46^. In the present study, the increase in noradrenergic activity in LC significantly elevated norepinephrine levels in hypothalamus and VLPO. These two areas are critical for sleep-wake states and neuropsychological regulation^46^. Norepinephrine and dopamine levels were elevated in VLPO, suggesting that our hypothesis of decreased activity in VLPO might be secondary to the activation of noradrenergic neurons in LC.

Presumed sleep-promoting neurons are GABAergic, galaninergic, and multi-polar triangular-shaped and have a potent low-threshold calcium potential^47^. These neurons are always inhibited by norepinephrine. Other studies have reported that postsynaptic α_2_-adrenoceptors mediate this effect^48^. When VLPO neurons begin to fire at sleep onset, they inhibit wake-promoting neurons, allowing for their own disinhibition and reinforced firing. Conversely, during arousal, wake-promoting neurons fire at a high rate, thus inhibiting VLPO neurons. The reciprocal inhibitory interaction between these systems provides a mechanism for the maintenance of one of two stable configurations. Accordingly, the disruption of wake- and sleep-promoting pathways results in behavioral instability caused by destabilization of reciprocal inhibitory interactions. The functional properties of the “reciprocal inhibitory interaction” model support the production of a stable waking state by a simple neuronal network and important resistance to behavioral switching by limiting inappropriate changes when inputs to VLPO or wake-promoting areas fluctuation. Our results showed that increased LC activity suppressed VLPO activation, and eventually inhibited the transition from wake to sleep, which was represented by prolonged the sleep latency and decreased sleep time.

The present study showed that repeated CORT treatment significantly increased REM sleep. Previous reports found no changes^18^ or reduction of REM sleep^7^,^49^ produced by acute administration of glucocorticoids. These discrepancies may due to the different doses and duration of glucocorticoids treatment, as well as different sleep recording period and species.

In conclusion, our findings indicate that GRs in LC may play an important role in chronically elevated CORT-induced sleep disorders through the activation of noradrenergic LC neurons. Increased TH and norepinephrine levels led to the suppression of GABAergic VLPO neurons and consequently sleep disorders. A GR antagonist, which has been previously reported to have antidepressant effect in animal studies^50^, also has important effect on sleep-wake regulation, particularly when circulating corticoids were above physiological levels, and may prompt the development of therapeutic strategies for stress-related sleep disorders. However, the underlying mechanism of the ability of GRs to regulate sleep requires further investigation.

## Methods

### Animals and treatment

Male Sprague-Dawley rats (270–300 g, Grade I, purchased from the Animal Center of Peking University, Beijing, China) were used. All experiments were conducted in accordance with the European Community guidelines for the use of experimental animals and approved by the Peking University Committee on Animal Care and Use. The rats were individually housed in acrylfiber cages under a 12 h/12 h light/dark cycle (lights on at 9:00 AM) and had *ad libitum* access to food and water. The ambient temperature was 23 ± 1°C, and the relative humidity was 50 ± 10%.

### Drugs and drug administration

According to the previous studies^51^,^52^, rats were injected subcutaneously with 40 mg/kg CORT (corticosterone 21-acetate, Sigma, St. Louis, MO, USA) dissolved in saline with 2% Tween 80 (Sigma, St. Louis, MO, USA) continuously for 7 days at 9:00 AM. In the microinjection experiment, RU486 (Sigma, St. Louis, MO, USA) was used as GR antagonist as previously reported^53^ and microinjected into LC in a volume of 0.25 μl/side 30 min before CORT administration every day at doses of 100 ng/side and 250 ng/side by dissolving in saline with 50% ethanol.

### Surgery and sleep analysis

At least 1 week prior to sleep recordings, the animals (9 for vehicle and 8 for CORT group) underwent surgery using previously described standard procedures^54^. Briefly, under chloral hydrate (300 mg/kg) anesthesia, two stainless steel screws attached to insulated wire were implanted in the skull over the frontal-parietal cortex for electroencephalography (EEG). One of the screws was placed approximately 2 mm anterior and 2 mm to the right of bregma, and the other screw was placed approximately 3 mm posterior and 2 mm to the left of bregma. A ground electrode was placed between the two screws, 3 mm lateral to the midline. For electromyography (EMG), a pair of wire electrodes was threaded through the nuchal muscles. These electrodes were attached to a miniature connector that was fixed to the skull with dental acrylic. After surgery, the rats were injected with penicillin for 3 days and allowed to recover for 7 days prior to the experiments. For habituation, they were connected to the recording apparatus at least 1 day before the sleep recording.

For the electrophysiological recordings, all rats were placed in an electrically shielded box in a noise-attenuated environment with a light-weight shielded cable plugged into the connector on the rat's head and attached to a counterbalanced swivel. The signals were routed to an electroencephalograph (model MP 150, BIOPAC Systems, CA, USA). Recordings were performed for 6 h, beginning at 9:00 AM, immediately after the last CORT injection. The signals were amplified and filtered (EEG, 0.5–30 Hz; EMG, 10–100 Hz) and then digitized at a sampling rate of 128 Hz and recorded using AcqKnowledge software (BIOPAC Systems). The EEG/EMG recordings were subjected to manual scoring using SleepSign 2.0 software (BIOPAC Systems), with the following criteria^55^: wakefulness (low-amplitude EEG activity and high-voltage EMG activity), rapid-eye-movement (REM) sleep (Fast-fourier transform [FFT] theta ratio of EEG ≥ 60%, desynchronized EEG, absence of tonic EMG, and occasional body twitches while maintaining a recumbent sleep posture), slow-wave sleep (SWS; FFT delta ratio of EEG ≥ 70%, large-amplitude, synchronous EEG with sleep spindles present, greatly diminished tonic EMG, eyes closed and recumbent posture), and light sleep (FFT delta ratio of EEG < 70%, high-amplitude slow or spindle EEG activity, and low-amplitude EMG activity). Non REM (NREM) sleep time was equal to SWS time + light sleep time. The total sleep time was equal to NREM sleep time + REM sleep time.

### Tissue preparation and immunostaining

Another set of animals were perfused through the ascending aorta with 0.9% NaCl solution 1 h after the last injection of CORT on day 7, followed by 4% paraformaldehyde in 0.1 M phosphate buffer^56^. Whole brains were immediately removed and post-fixed in the same fixative at 4°C for 24 h and then immersed in 30% sucrose in 0.01 M phosphate-buffered saline (PBS; pH 7.4) at 4°C for cryoprotection. Prior to tissue sectioning, the brains were blocked at approximately +1.0 mm with reference to bregma, rapidly frozen in liquid n-hexane that was cooled with a mixture of solid carbon dioxide and ethanol, and stored at −20°C.

Coronal sections that encompassed VLPO, Pef, TMN, PPT, DRN, LDT, and LC were freeze-cut at 20 μm thicknesses. The anatomical nomenclature (based on Paxinos and Watson, 1998) and protocol are summarized in Table 2. Each section was first immunostained for nucleus-specific neurotransmitter markers (Table 2) and then processed for Fos staining.

### Cell counting

For each animal, two double-immunostained sections were selected for each rostrocaudal level that was counted. Each section was assessed with regard to the number of Fos-immunopositive/neuron-immunonegative (Fos+/neuron−) nuclei, total number of neuron-immunopositive (Fos±/neuron+) cells (both Fos+ and Fos−), and number of double-immunostained (Fos+/neuron+) cells. The double-stained cell ratio (i.e., the number of Fos+/neuron+ cells divided by the number Fos±/neuron+ cells, multiplied by 100%) was compared in the different treatment groups. Black immunostaining was counted as Fos+ nuclei, and brown staining throughout the cytoplasm was counted as neuron+ cells. Cells with brown staining of the cytoplasm and black staining of nuclei were counted as double-immunostained cells.

### Western blot

Based on the previous studies^57^,^58^, VLPO and LC tissue (with some of their surrounding tissue) were punched (1 mm diameter for VLPO and 2 mm diameter for LC) bilaterally using brain matrix guided by the rat brain atlas of Paxinos and Watson (1998). VLPO and LC proteins were pooled, extracted, boiled in 1% sodium dodecyl sulfate (SDS) solution, and quantified using a BCA assay kit (Pierce, Rockford, IL, USA) with bovine serum albumin as the standard. Equal amounts of protein (25 μg) were separated by 10% SDS-polyacrylamide gel electrophoresis (PAGE) and transferred to polyvinylidene difluoride membranes (Millipore, MA, USA). The membranes were blocked with 5% non-fat milk for 1 h at room temperature and incubated with primary antibodies, including anti-β-actin (1:1000; Abmart, Shanghai, China), anti-histone H3 (1:2000; Cell Signaling Technology, MA, USA), anti-GAD (1:1000; Chemicon Millipore, MA, USA), anti-TH (1:1000; Santa Cruz Biotechnology, Santa Cruz, CA, USA), anti-GR (1:1000; Santa Cruz Biotechnology, Santa Cruz, CA, USA), and anti-MR (1:100; Santa Cruz Biotechnology, Santa Cruz, CA, USA) in TBS-T buffer (Tris-buffered saline +0.1% Tween-20) at 4°C overnight. After 3 × 10 min TBS-T washes, the blots were incubated with horseradish peroxidase-conjugated secondary antibodies (1:1000; Abmart Shanghai, China) for 2 h at 37°C and then washed with TBS-T buffer for 3 × 10 min. The blots were then treated with an enhanced chemiluminescence detection kit (CoWin Bioscience, Beijing, China). Western blot bands were scanned with a GelDoc XR System (Bio-Rad, Hercules, CA, USA) and subsequently analyzed densitometrically using Image Lab software. The results were normalized to the protein expression level of β-actin or histone in each sample.

### Microinjection into locus coeruleus

Together with the EEG/EMG surgery described above, additional steps were performed as described below. Rats were anesthetized and positioned in a stereotaxic instrument. A double guide cannula (26 gauge, C/C dist. 2.4 mm, Plastics One, Roanoke, VA) was implanted with the tip 1 mm above the LC (anterior/posterior, −9.8 mm; lateral, ±1.2 mm; dorsal/ventral, −6.0 mm below the brain surface). The microinjections were performed with a Hamilton syringes that were connected to the 33 gauge injection cannula (Plastics One, Roanoke, VA). Drug or vehicle was delivered through the injection cannula that extended 1 mm beyond the guide in a 0.25 μl volume over a 2-min period. The injection cannula was kept in place for another 2 min to allow the drug to completely diffuse from the tip. At the end of the experiments, cannula placements were defined histologically using Nissl staining with thickness of 20 μm under light microscopy. The correctness of the cannula/injection sites was assessed according to the atlas of Paxinos and Watson (1998). All the data presented in this report are derived from animals whose injection site was within the limits of the LC. The location of injection tip is showed in Fig. 4a.

### High-performance liquid chromatography analtsis

According to previous report^59^, the VLPO, LC, hypothalamus, and prefrontal cortex were dissected and extracted with 0.2 M perchloric acid by ultrasonic homogenation. The mixture was centrifuged twice at 14,000 rpm for 50 min at 4°C. The levels of norepinephrine and dopamine were detected by high-performance liquid chromatography with electrochemical detection (HPLC-ECD; Dionex) using a C18 reverse-phase column (3.0 mm inner diameter, 75 mm length; Capcell Pak C18 MG S3, Shiseido). The mobile phase of the HPLC system consisted of 0.85 mM sodium octylsulfate, 0.5 mM ethylenediamine tetraacetic acid, and 0.1 mM NaH_2_PO_4_ in 11% methanol, pH 3.25. The flow-rate was set at 0.6 ml/min. The electrochemical detector was set with an oxidizing potential of 200 mV and reducing potential of −175 mV.

### Statistical analysis

The data were analyzed using SPSS software 17.0 (SPSS Inc., Chicago, IL, USA) and are expressed as mean ± SEM. The biochemical data for comparisons between the vehicle- and CORT-treated groups were analyzed using unpaired Student's *t*-test. One-way analysis of variance followed by the Student-Newman-Keuls *post hoc* test was used for multiple comparisons. In all of the tests, *p* < 0.05 was considered statistically significant.

## Author Contributions

Y.H.Z. and Z.J.W. conceived and designed the experiments. Z.J.W., X.Q.Z., B.Y., Z.F.S., S.J.L., Y.L.H., Q.C., Y.P.X., X.Y.C. and S.Y.C. performed the experiments and analysed the data. Y.H.Z. and Z.J.W. wrote the manuscript. X.Q.Z. edited the manuscript.

## Figures and Tables

**Table 1:** Treatment of CORT (40 mg/kg, s.c.) for 7 days altered sleep parameters in rats

	Vehicle	CORT (40 mg/kg, s.c., 7 days)
Total sleep (min)	247.2 ± 6.6	198.0 ± 3.8**
NREM sleep (min)	230.2 ± 6.5	170.1 ± 4.6**
Light sleep (min)	227.7 ± 6.5	169.4 ± 7.2**
Slow wave sleep (min)	8.0 ± 2.9	4.7 ± 1.6
REM sleep (min)	18.2 ± 1.5	27.8 ± 1.5*
Sleep latency (min)	25.9 ± 3.6	48.1 ± 4.1**
REM sleep latency (min)	32.7 ± 4.9	6.9 ± 1.2**
Light sleep (%)	92.1 ± 3.7	85.6 ± 3.5
Slow wave sleep (%)	3.3 ± 1.2	2.3 ± 0.8
REM sleep (%)	7.3 ± 0.5	14.0 ± 1.8**

Data are represented as mean ± S.E.M. (*n* = 8 ~ 9/group); *p < 0.05 and **p < 0.01 *vs* vehicle (Student's *t*-test).

**Table 2:** The anatomical nomenclature and protocol in immunohistochemistry

Brain area	Coronal to bregma (mm)	Specific protein*	Source of primary antibody Catalog number	Primary antibody (12 h at 4°C)	Secondary antibody system Catalog number*	Develop solution and color
VLPO	−0.3 ~ −0.92	GAD	MAB-5406 Chemicon Millipore	1:1000	HRP Detection System (PV-9005)	DAB (brown)
Pef	−2.8 ~ −3.6	Orexin	PC-345 Chemicon Millipore	1:50	HRP Detection System (PV-9001)	DAB (brown)
TMN	−3.8 ~ −4.52	ADA	AB-176 Chemicon Millipore	1:3000	HRP Detection System (PV-9001)	DAB (brown)
PPT	−6.72 ~ −8.3	ChAT	MAB-305 Chemicon Millipore	1:1000	HRP Detection System (PV-9005)	DAB (brown)
DRN	−7.04 ~ −8.3	TrpOH	AbD, 9260-2505	1:1000	HRP Detection System (PV-9003)	DAB (brown)
LDT	−8.72 ~ −9.3	ChAT	MAB-305 Chemicon Millipore	1:500	HRP Detection System (PV-9005)	DAB (brown)
LC	−9.16 ~ −10.52	TH	22941, ImmunoStar Inc	1:2000	HRP Detection System (PV-9005)	DAB (brown)
all		c-Fos	Calbiochem, PC38T Merck	1:1000	HRP Detection System (PV-9001)	DAB-Ni (black)

*GAD: Glutamic acid decarboxylase; ADA: Adenosine deaminase; ChAT: Choline acetylase; TrpOH: Tryptophan hydroxylase; TH: Tyrosine Hydroxylase.

*All the secondary antibody systems were purchased from GBI, USA.
